# The Chemokine Receptor CCR3 Is Potentially Involved in the Homing of Prostate Cancer Cells to Bone: Implication of Bone-Marrow Adipocytes

**DOI:** 10.3390/ijms22041994

**Published:** 2021-02-17

**Authors:** Adrien Guérard, Victor Laurent, Gaëlle Fromont, David Estève, Julia Gilhodes, Edith Bonnelye, Sophie Le Gonidec, Philippe Valet, Bernard Malavaud, Nicolas Reina, Camille Attané, Catherine Muller

**Affiliations:** 1 Institut de Pharmacologie et de Biologie Structurale (IPBS), Université de Toulouse, CNRS UMR 5089, 205 Route de Narbonne, 31077 Toulouse, France; adrienguerard@wanadoo.fr (A.G.); Victor.Laurent@evotec.com (V.L.); david.esteve@ipbs.fr (D.E.); 2 Service d’Anatomie Pathologique, CHRU de Tours, Inserm UMR 1069, 37000 Tours, France; gaelle.fromont-hankard@univ-tours.fr; 3 Département de Biostatistiques, Institut Universitaire du Cancer, CEDEX 9, 31059 Toulouse, France; Gilhodes.Julia@iuct-oncopole.fr; 4 Lyos, INSERM, UMR1033, 69008 Lyon, France; Edith.Bonnelye@univ-nantes.fr; 5 Institut des Maladies Métaboliques et Cardiovasculaires (I2MC), INSERM U1048, Université de Toulouse, 31432 Toulouse, France; sophie.le-gonidec@inserm.fr (S.L.G.); Philippe.Valet@inserm.fr (P.V.); 6 Département d’Urologie, Institut Universitaire du Cancer, CEDEX 9, 31059 Toulouse, France; bernard.malavaud@me.com; 7 Service de Traumatologie et de Chirurgie Orthopédique, CHU de Toulouse—Purpan, 31059 Toulouse, France; reina.n@chu-toulouse.fr

**Keywords:** prostate cancer, bone metastasis, bone marrow adipocytes, CCR3, chemokine

## Abstract

Bone metastasis remains the most frequent and the deadliest complication of prostate cancer (PCa). Mechanisms leading to the homing of tumor cells to bone remain poorly characterized. Role of chemokines in providing navigational cues to migrating cancer cells bearing specific receptors is well established. Bone is an adipocyte-rich organ since 50 to 70% of the adult bone marrow (BM) volume comprise bone marrow adipocytes (BM-Ads), which are likely to produce chemokines within the bone microenvironment. Using in vitro migration assays, we demonstrated that soluble factors released by *human* primary BM-Ads are able to support the directed migration of PCa cells in a CCR3-dependent manner. In addition, we showed that CCL7, a chemokine previously involved in the CCR3-dependent migration of PCa cells outside of the prostate gland, is released by *human* BM-Ads. These effects are amplified by obesity and ageing, two clinical conditions known to promote aggressive and metastatic PCa. In *human* tumors, we found an enrichment of CCR3 in bone metastasis vs. primary tumors at mRNA levels using Oncomine microarray database. In addition, immunohistochemistry experiments demonstrated overexpression of CCR3 in bone versus visceral metastases. These results underline the potential importance of BM-Ads in the bone metastatic process and imply a CCR3/CCL7 axis whose pharmacological interest needs to be evaluated.

## 1. Introduction

Prostate cancer (PCa) is a major healthcare problem, hitting the third rank in term of mortality by cancer in men and the first rank in term of incidence [1]. Dissemination of the tumor cells outside of the prostate gland within the proximal fat depot (called Peri Prostatic Adipose Tissue, PPAT) is a widely acknowledged adverse factor in PCa prognosis [2]. Cell migration is an essential component of metastatic dissemination of tumor cells from the primary tumor to local and distant sites. Invasion and dissemination are most efficient when the cells are involved in directed migration that involve chemokines and their receptors [3]. We have previously demonstrated that adipocytes from PPAT favor the initial step of PPAT infiltration by secreting the CCL7/MCP3 chemokine that attracts CCR3-expressing cancer cells, and this process is amplified in obesity [4]. Indeed, obesity, a condition where the secretory profiles of PPAT is modified, is associated with greater occurrence of developing advanced and high-grade PCa [5,6]. In *human*, expression of the C-C chemokine receptor CCR3 is associated with the occurrence of aggressive disease with extended local dissemination and a higher risk of biochemical recurrence [4]. In addition to PCa, recent studies demonstrate that this receptor is involved in thyroid [7], colon cancer [8] and glioblastoma [9] dissemination. Hitherto, the central role of this receptor was demonstrated in eosinophil trafficking in allergic airway diseases leading to the development of targeted inhibitors [10]. 

Even if local dissemination is a crucial step in PCa progression, the prognosis of PCa is determined by the presence of metastases, that will be, in about 80 percent of the time, into the bones [11]. Multiple chemokines and their receptors have been implicated in PCa progression [12,13], but the picture lacks clarity when the question of bone homing is raised and the role of CCR3 has never been investigated in this context [14]. It has been suggested that disseminated tumor cells can use the same physiological mechanisms as those used by hematopoietic stem cells (HSCs) homing to bone, involving primarily the CXCR4/CXCL12 axis [15]. In animal models, metastatic PCa directly compete for the occupancy of the HSC niche during localization to the bone marrow (BM) [16]. In the BM microenvironment, CXCL12 is primarily expressed by perivascular mesenchymal stromal cells and at lower levels by endothelial cells and osteoblast [17]. Pre-treatment of PCa cell lines with anti-CXCR4 antibodies or use of CXCR4 antagonist decreases their ability to form bone metastasis in a *mouse* model [18,19]. At clinical levels, data are clearly lacking. A recent review of the literature suggests that the increased expression of CXCR4 in primary tumors is significantly associated with lymph nodes or bone metastasis occurrence in *human* [20]. Besides the CXCR4/CXCL12 axis, other studies have implicated the CCL2/CCR2 axis in vitro and in animal models, although the experimental design used in these studies more specifically addressed tumor growth into bones rather than homing from distant sites [21]. According to our results involving CCR3 in local PCa dissemination along a concentration gradient of adipocytes-secreted chemokines, we asked whether this receptor might also be involved in the directed migration of PCa cells to bones through the involvement of bone-marrow adipocyte (BM-Ad) secretion. 

Bone is an adipocyte-rich organ since 50 to 70% of the adult bone marrow (BM) volume comprise BM-Ads [22]. As adipocytes from other depots, BM-Ads appear to secrete bioactive molecules termed adipokines, but limited data are available concerning their secretory profile and its functional implication [22,23]. The number and size of adipocytes in the BM increase with obesity as well as during ageing, conditions compromising hematopoiesis as well as bone formation [24,25]. Obesity and ageing have also been associated with increased bone metastasis in PCa, suggesting a potential role of BM-Ads in this process [26,27]. Therefore, these two conditions, obesity and ageing, share common features. We have recently set up the purification of *human* primary BM-Ads and demonstrated these cells exhibit a very specific lipid metabolism as compared to subcutaneous adipocytes (SC-Ads) [28]. The use of *human* material is of major importance since specie-specific differences exist between rodent and *human* BM adipose tissue [22,23]. In addition, our recent study highlights the importance of using primary *human* BM-Ads since their phenotype are not recapitulated by *human* BM mesenchymal cells (BMSCs) differentiated in vitro [28]. Our current work first demonstrated that in vitro *human* primary BM-Ads secretions are able to support PCa directed migration in a CCR3-dependent manner. In addition, we demonstrated that, in *human* samples, PCa bone metastasis were enriched in CRR3 positive cells. Taken together, the results obtained support a potential role for CCR3 in the homing of PCa cells to bone. 

## 2. Results 

### 2.1. Obesity and Ageing Increase the Ability of BM-Ads to Promote the Migration of Cancer Cells 

**Figure 1:**
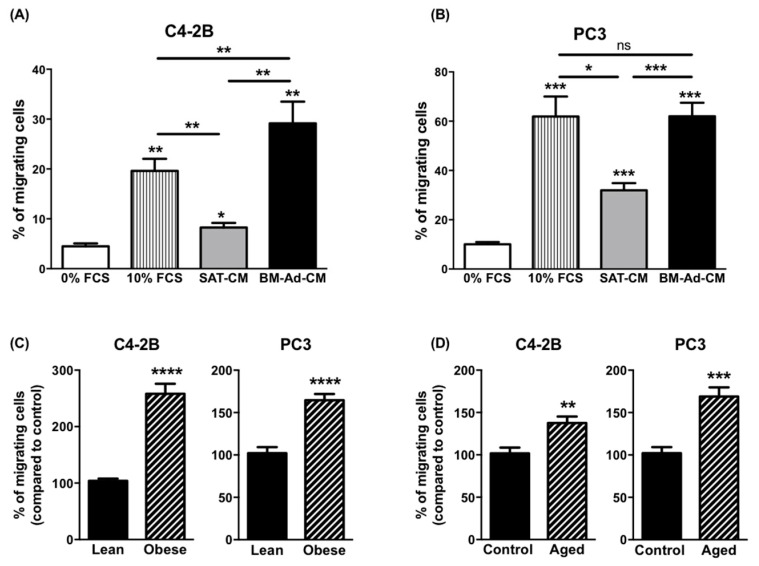
The chemotaxis of PCa cells towards BM-Ad-CM is enhanced by obesity and ageing. In vitro migration of C4-2B (**A**) or PC3 (**B**) towards a medium containing either 0% (used as a negative control), 10% FCS or towards SAT-CM or BM-Ad-CM (*n* = 4–5/group). Statistical analysis by One-way ANOVA with Holm-Sidak’s multiple comparisons test was performed. (**C**) In vitro migration of the indicated PCa cell lines towards BM-Ad-CM from lean (age: 62.8 ± 6.9 years, BMI: 21.4 ± 3.3 kg/m^2^) or obese (age: 63.3 ± 10.1 years, BMI: 33.3 ± 2.3 kg/m^2^) subjects (*n* = 5/group). (**D**) Similar experiments were performed with aged (age: 75.7 ± 5.7 years, BMI: 23.3 ± 4.3 kg/m^2^) and younger control (age: 60.2 ± 5.2 years, BMI: 22.1 ± 3.5 kg/m^2^) subjects (*n* = 5/group). Data are shown as mean ± sem (**A**,**B**) or mean of migrating cells (with the control groups set at 100%) ± sem (**C**,**D**). Statistical analysis by Student’s t-test was performed. * *p* < 0.05, ** *p* < 0.01, *** *p* < 0.001, **** *p* < 0.0001, ns: not significant.

To study the chemoattractant potential of BM-Ads, conditioned media (CM) were prepared from either BM-Ads or paired subcutaneous adipose tissue (SAT) obtained from male patients (age between 51 and 73 years and with body mass index (BMI) between 20 and 25 kg/m^2^). Directed migration assays were performed on two PCa cell lines with low (C4-2B) or high (PC-3) aggressiveness (Figure 1A,B). The ability of BM-Ads to chemoattract tumor cells was at least equivalent (PC-3 cells) or greater (C4-2B) than the 10% FCS positive control whereas SAT-CM was significantly less chemoattractant than BM-Ad-CM.

As obesity and ageing have been associated to increased distant dissemination in PCa [26,29], similar experiments were performed with samples obtained from obese (average BMI 33.3 ± 2.3 vs. 21.4 ± 3.3 kg/m^2^ in obese versus control group) or elderly (average age: 75.7 ± 5.7 vs. 60.2 ± 5.2 years in aged versus younger control group) patients. In obese condition, the ability of BM-Ad-CM to chemoattract tumor cells was increased for C4-2B and PC3 by about 2.6- and 1.7-fold respectively as compared to the control group (Figure 1C). A slight effect of obesity was observed with SAT-CM, but this effect was less pronounced than with BM-Ad-CM (Supplementary Figure S1A,B). Similarly, the chemoattractive potential of BM-Ad-CM from aged patients was increased in PCa cell lines as compared to the younger control group (Figure 1D) and this effect was lower with SAT-CM (Supplementary Figure S1C,D).

### 2.2. CCR3/CCL7 Axis Is Involved in the Directed Migration of PCa Cells towards BM-Ad-CM 

**Figure 2:**
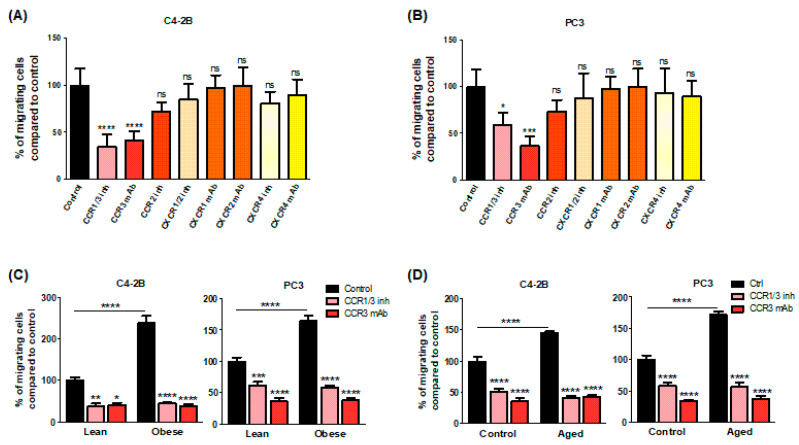
CCR3 is an important driver in the directed migration of PCa cells towards BM-Ad-CM and its effect is majored in obesity and ageing conditions. In vitro migration of C4-2B (**A**) or PC3 (**B**) Table 1. CCR3 (UCB35625, 200 nM), CCR2 (sc-202525, 25 nM), CXCR1/2 (SB225002 inhibitor, 50 nM) or CXCR4 (AMD3100, 100 nM) or with mAbs against CCR3, CXCR1, CXCR2 or CXCR4 (all used at 10 μg/ml). Bar plots represent the percentage of migrating cells relative to the migration of untreated cells (set to 100%). Data are shown as mean ± sem (*n* = 4–6). The statistical significance between means of migrating cells (in %) in treated vs. control cells was evaluated by One-way ANOVA with Tukey’s multiple comparisons test. Similar experiments were performed with the indicated cell lines towards BM-Ad-CM obtained either from lean/obese (**C**) or control/aged (**D**) subjects in the presence of CCR3 inhibitors and blocking mAb. Data are shown as mean ± sem (*n* = 4–9). The statistical significance between mean of migrating cells (with the control groups set at 100%) was evaluated by Two-way ANOVA with Sidak’s multiple comparisons test. * *p* < 0.05, ** *p* < 0.01, *** *p* < 0.001, **** *p* < 0.0001, ns: not significant.

**Figure 3:**
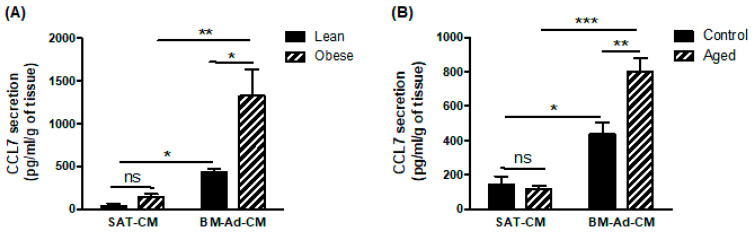
CCL7 is highly secreted by BM-Ads and its secretion is enhanced in obesity and ageing conditions. Secretion of CCL7 was evaluated by ELISA in SAT-CM or BM-Ad-CM either obtained from lean/obese (**A**) or control/aged (**B**) subjects. Data are shown as mean ± sem (*n* = 3–5). The statistical significance was evaluated by Two-way ANOVA with Tukey’s multiple comparisons test. * *p* < 0.05, ** *p* < 0.01, *** *p* < 0.001, ns: not significant.

To investigate which chemokine receptor is involved in the chemoattractant potential of BM-Ads, both antagonists and blocking mAbs were used in the directed migration assays. UCB35625 is a dual inhibitor of CCR1/CCR3, but we have previously demonstrated that the PCa cell lines do not express the CCR1 receptor [4]. UCB35625 as well as CCR3 neutralizing mAb inhibit the migration of both PC3 and C4-2B cell lines by 40 to 60% (Figure 2A,B). By contrast, no significant effect was seen with antagonists or blocking mAbs directed against CCR2 (for which no validated commercial blocking antibody was available), CXCR1, CXCR2 and CXCR4 (Figure 2A,B). We then investigated the regulation of these effects in obese or elderly subjects. Inhibition of CCR3 completely abrogated the increase of cell migration observed in obesity (Figure 2C) and during ageing (Figure 2D) in both PC3 and C4-2B cell lines. We have previously demonstrated that CCL7 is the main CCR3 ligand regulated by obesity in PPAT [4]. Similarly, CCL7 secretion is significantly enhanced in BM-Ad-CM from obese or elderly subjects (Figure 3A,B). CCL7 secretion was significantly increased in SAT-CM in obese versus lean patients whereas no effect of ageing was observed (Figure 3A,B). These results suggest that obesity and ageing increased the directed migration of PCa cells modulating the secretory pattern of adipocytes, this effect being mainly dependent on the CCR3/CCL7 axis.

### 2.3. Expression of CCR3 Is Increased at Bone Metastatic Sites of Human PCa 

**Figure 4:**
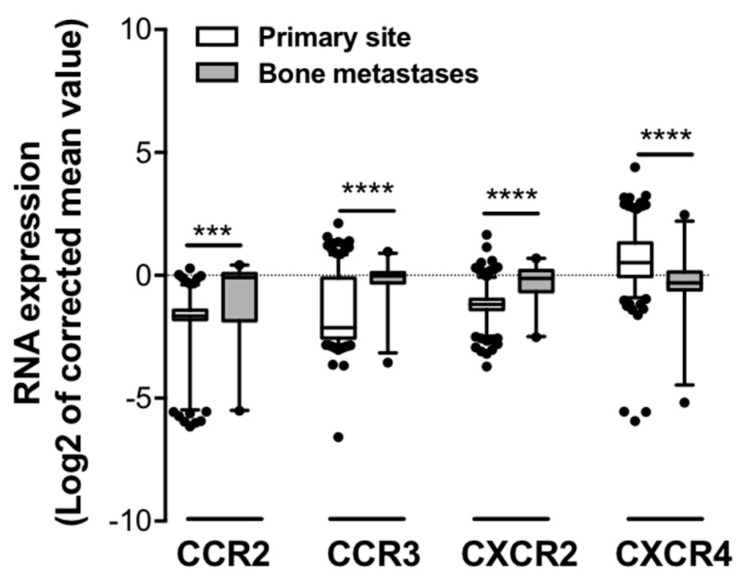
Expression of chemokine receptors in primary versus bone metastases of *human* PCa. The cancer microarray database, Oncomine was used to compare the RNA expression of several chemokine receptors (CCR2, CCR3, CXCR2 and CXCR4) in patient samples collected from bone metastatic (grey squares) or primary sites (white squares) from six different data-sets. Differences between the two independent groups were evaluated with Mann-Whitney’s test. *** *p* ≤ 0.001, **** *p* ≤ 0.0001.

Our in vitro results demonstrate that BM-Ads support PCa cell directed-migration, this effect being dependent of the CCR3/CCL7 axis. These results support the hypothesis that CCR3 might be involved in the homing of PCa cells to bone. To investigate this hypothesis, we explore whether the *human* bone metastatic sites were enriched with tumor cells expressing CCR3 using two different strategies. First, the cancer microarray database Oncomine was used to identify microarray datasets comparing the expression of genes in primary tumors versus distant bone metastases in order to create a specific cohort suitable for meta-analysis. The expression pattern of CCR3 was investigated along with those of CCR2, CXCR2 and CXCR4 that have been previously involved PCa bone metastases in pre-clinical studies [4,20,21]. Six datasets were identified which contained the chosen chemokines receptor expression data including overall 277 samples with 249 primary tumors and 28 bone metastatic sites of PCa (Table 1). Using this cohort, we showed that the expression of CCR3, as well as CCR2 and CXCR2, was up-regulated in bone metastatic sites compared to primary tumors whereas CXCR4 expression was down-regulated (Figure 4). Therefore, this first data set links CCR3 expression to PCa bone metastases in human.

**Figure 5:**
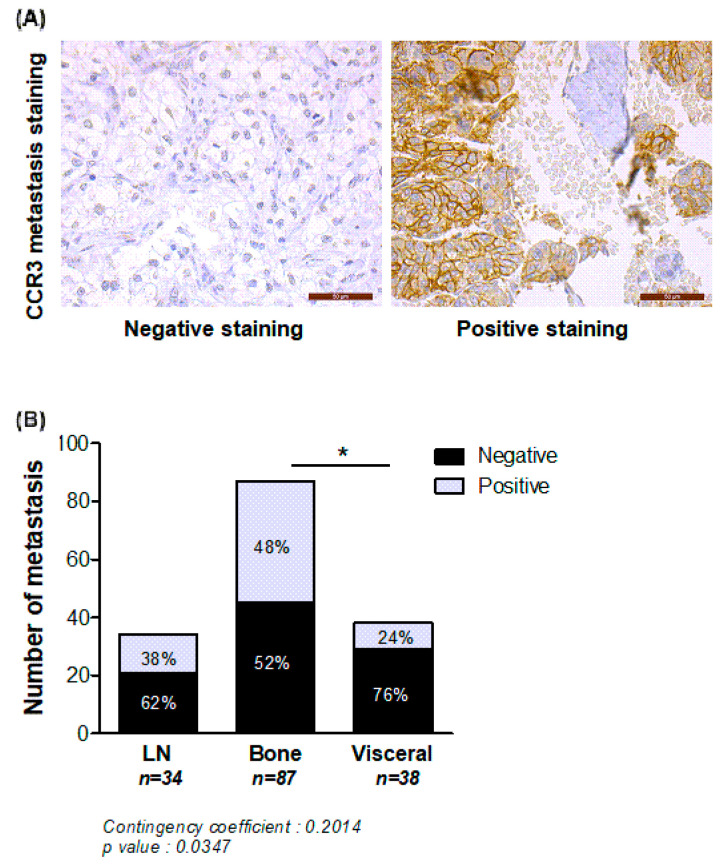
CCR3 is overexpressed in bone compared to visceral metastasis in *human* PCa. Metastatic PCa samples coming from different sites (lymph nodes (LN), visceral or bone) were stained for CCR3, and categorized as positive or negative for CCR3 expression. (**A**) Representative examples of negative and positive staining in bone metastases are shown. Scale bars, 50 μm. (**B**) Quantification of the number of metastasis negative or positive for CCR3 according to their anatomical location (LN (*n* = 34), visceral (*n* = 38) or bone (*n* = 87). Percentage of each group is mentioned in each bar graph. Chi2 statistic test was performed (*p* = 0.0347) and contingency coefficient was calculated (0.2014). Comparison between location was evaluated by fisher’s exact post-test (* *p* < 0.05).

Secondly, to strengthen the clinical relevance of our results, we investigated CCR3 expression in different metastatic sites of PCa in two independent TMA. As shown in Figure 5, the number of CCR3 positive tumors was significantly higher in bone versus visceral metastasis, whereas no significant differences were observed between lymph nodes and bone sites. It is interesting to note that the majority of visceral metastases were negative for CCR3 expression. However, the fact that 50% of the bone metastasis do not express CCR3 suggest that other chemokine receptors might also be involved in this process. 

## 3. Discussion

Our study demonstrates, for the first time, the implication of CCR3 in the in vitro directed migration towards BM-Ad secretions and the enrichment of CCR3 expressing cells in *human* bone metastatic sites, suggesting that this pathway could be involved in the homing of PCa cells to bone. 

Using conditioned medium from *human* primary BM-Ads, we first demonstrated that their secretions possess a strong ability to support the directed migration of PCa cells. The localization of tumors towards adipocyte-rich microenvironment during metastatic process has been already described (for review [36,37]). For example, ovarian cancer cells preferentially metastasize to the omentum (a large intra-peritoneal fat pad) [38]. Leukemic cells also disseminate to SAT and visceral adipose tissues (VAT) at least in mouse models [39,40]. Using a set of blocking mAbs and pharmacological inhibitors, we demonstrate that CCR3 is a master regulator of PCa cell migration towards BM-Ad-CM. By contrast, metastases of ovarian cancer to omentum involve predominantly CXCR1 [38] and CXCR4 is necessary for leukemic cells dissemination to SAT and VAT [40]. These results highlight that the homing of tumor cells to adipose tissue could be dependent on both tumor-and host-dictated (nature and anatomical location of the adipose depots) characteristics. For PCa, the migration towards both surrounding (PPAT) [4] or distant adipose tissue (BM-adipose tissue) is dependent of CCR3 suggesting the predominance of tumor type over depots. 

As for PPAT [4], we found that BM-Ads secrete the chemokine CCL7. Among the CCR3 ligands, CCL7 has been recently implicated in the dissemination of several cancers such as the dissemination of colon and lung cancers to liver [8,41,42]. To our knowledge, the pattern of the chemokines secreted by primary *human* BM-Ads has never been studied while some results were obtained using BMSCs differentiated in vitro in adipocytes. In *human*, in vitro BMSC-derived adipocytes secrete the cytokines IL-6, MIP-1/CCL3, G-CSF, and GM-CSF [43] and in mice, they also produce CXCL1 and CXCL2 [44], both ligands of the CXCR2 receptor. However, we recently showed that BMSCs differentiated in adipocytes in vitro do not recapitulate the phenotype of primary BM-Ads [28] and results obtained with these models should be interpreted with caution. To our knowledge, our study is the first to report in vitro directed migration of tumor cells towards primary *human* BM-Ad secretions. In addition, we observed that CCL7 secretion by BM-Ads was up-regulated by obesity and during ageing; an increased CCL7 secretion by PPAT of obese subjects having also been demonstrated in our previous study [4]. Secretion of chemokines is upregulated in obesity mainly in VAT from obese animal models and *humans* [45] whereas this regulation has not been studied in the context of ageing so far. Ageing is associated with the occurrence of a senescent phenotype in mature adipocytes marked by the overexpression of beta-galactosidase, increased expression of p53 and P16^INK4a^ and increased ROS generation [46]. This senescent phenotype is associated with an increased proinflammatory cytokines secretion as well as metabolic dysfunctions characterized by altered lipolysis and decrease glucose uptake [46]. These results have been obtained in mature adipocytes from SAT and VAT and to our knowledge, the characterization of phenotypical changes induced by ageing in *human* BM-Ads has never been performed. In our study, the observed increase in migration associated with obesity and ageing is totally abrogated when CCR3 is inhibited. Taken together, our results show that obesity and ageing increased the directed migration of PCa cells by modulating the secretory pattern of adipocytes, this effect being dependent on the CCR3/CCL7 axis. Interestingly, adipose tissue senescence is one of the common features in obesity and ageing [46] and our preliminary results suggest that these two conditions might similarly affect the secretory profile of BM-Ads. 

Finally, a set of data collected in *humans* potentially highlights the clinical relevance of our study. Having demonstrated the role of CCR3 in in vitro directed migration, we evaluated if the bone metastatic sites were enriched with tumor cells expressing this receptor. The over-expression of CCR3 was demonstrated in bone metastasis vs. primary tumors using the cancer microarray database, Oncomine, and at the protein levels in bone vs. visceral metastasis by immunohistochemistry. Unfortunately, since the datasets used were not annotated in terms of metabolic parameters (even BMI), we were not able to demonstrate in *human*, the influence of obesity and ageing in this process. Although these results suggest that CCR3 favors the homing of PCa cells to bones, we cannot formerly exclude that cancer cells began expressing CCR3 once they have reached the bone in response to the bone environment. Our results showing a decreased *CXRC4* expression in bone metastasis using Oncomine are in apparent contradiction with the study of Chen and Zhong [20]. Several reasons can account for these discrepancies. Chen and Zhong evaluate the expression levels of *CXCR4* in primary tumors, which will further develop or not bone metastasis using therefore a correlative approach between *CXCR4* expression and aggressiveness. In contrast, we choose to compare the expression of chemokine receptors between primary sites and metastatic sites, meaning that the tumor cells have been able to successfully invade the bone. The fact that we used normalized RNA chip analysis also limit the inter-studies variation that could be observed in other meta-analysis.

In addition to our previous work [4], these results highlight that both local and distant dissemination of PCa might be dependent on CCR3. CCR3 appears to be a specific and interesting therapeutic target that could permit to limit bone metastases, the deadliest complication of PCa, but also help in controlling further development of the disease. Therefore, our results suggest new strategies for the treatment of advanced PCa, involving CCR3 antagonists, which are currently being developed for other diseases including asthma [10]. However, the involvement of CCR3 in bone metastasis remain to be firmly confirmed. Mouse models could be useful to answer this question [47]. However, in a homing process depending on the secretion of BM-Ads, there are several arguments suggesting that this might not be an appropriate model or at least will require extensive preliminary experiments to validate its appropriateness. Indeed, qualitative and quantitative differences exist between rodent and *human* BM-Ad highlighting that the results obtained in *mouse* may not reproduce the mechanisms existing in *humans* [23]. Two different types of BM-Ads have been described in *mouse*, regulatory and constitutive BM-Ads and their existence in *humans* remains unconfirmed [48]. Constitutive BM-Ads develop shortly after birth in the tail vertebrae and their number remain constant while regulatory BM-Ads develop postnatally within the BM of long bones, their number varies between *mouse* strains and some strains require pharmacological induction to obtain the presence of a significant number of BM-Ad [23]. Accordingly, the quantity of BM-Ads in rodents models remains much lower than in *humans* where BM-Ads consistently fills 50 to 70% of the bone marrow cavity of long bones [22]. 

Therefore, our study suggests that further investigation on the role of BM-Ads in cancer progression are clearly needed and especially in regard to their place in homing mechanisms and local progression of the bone lesions as they appear essential, but still neglected, cells.

## 4. Materials and Methods

### 4.1. Cell Lines and Culture

The *human* PCa cell lines C4-2B (from DSMZ, Braunschweig, Germany) and PC-3 (ATCC® CRL-1435™) were provided by Olivier Cuvillier; IPBS, Toulouse, France). Cells were cultured in RPMI medium supplemented with 10% fetal calf serum (FCS) and 1% streptomycin and penicillin (all purchased from GIBCO-Thermo Fisher Scientific, Eugene, OR, USA). All cell lines used in this study were grown in a humid atmosphere with 5% CO2 at 37 °C and were used within 2 months after thawing of frozen aliquots.

### 4.2. Human Subcutaneous Adipose Tissue (SAT) and Bone Marrow Adipocytes (BM-Ads) Conditioned Media Preparation

Paired *human* SAT and BM-Ads were collected from patient undergoing hip replacement surgery in the orthopedic surgery unit from Purpan hospital (Toulouse, France). Briefly, SAT and BM-Ads were collected as previously described [28]. All patients gave their inform consent and the procedure is approved by the French ministry of education and research (authorization DC-2017-2914; 31 May 2017). For the samples used for ageing studies, the age of the control group corresponds to the median of the lower half of the data set. BM-Ads were isolated by flotation and washed at least three times with DMEM medium supplemented with 1% BSA (Sigma-Aldrich, Saint Louis, MO, USA) and 1% penicillin and streptomycin (DMEM 1% BSA) to remove hematopoietic cells. SAT was cleaned of blood vessels and connective tissue and washed in DMEM 1% BSA. Conditioned media were obtained by incubating overnight 1 g of SAT or BM-Ads (SAT-CM or BM-Ad-CM respectively) in 8 mL DMEM 1% BSA.

### 4.3. Boyden Chamber Migration Assays

Cell migration assays were performed for 12h as previously described [3] using SAT-CM or BM-Ad-CM diluted to half in DMEM 1% BSA. As described in our previous study, the cell lines used expressed the chemokine receptors evaluated here (CCR3, CXCR1, CXCR2, CXCR4) [4]. When indicated, cells were pre-incubated for 30 min at 37 °C with 10 μg/mL blocking monoclonal antibodies (mAbs) directed against CCR3 (clone 444-11, reference D085-3, obtained from MBL International, Woburn, MA, USA), CXCR1 (Clone 42705, reference MAB330), CXCR2 (clone 48311, reference MAB331), CXCR4 (clone 44716, reference MAB172) or control IgG (all obtained from R&D Systems, Minneapolis, MN, USA). Cells were also pre-incubated for 30 min at 37 °C with pharmacological inhibitors: 200nM CCR1/CCR3 inhibitor, UCB35625 (Tocris, Bristol, UK), 25nM CCR2 inhibitor, sc-202525 (Santa Cruz Biotechnology, Dallas, TE, USA), 50nM CXCR1/2 inhibitor, SB225002 (Tocris, Bristol, UK) or 100nM CXCR4 inhibitor, AMD3100 (Sigma-Aldrich, Saint Louis, MO, USA). The doses used were defined in our precedent study [4].

### 4.4. PCa Tissue Micro Arrays (TMAs)

We used two TMAs to evaluate the expression of CCR3. The first one includes 39 visceral, 24 lymph node and 78 bone metastases from 45 castration resistant PCa patients and has been provided by the Prostate Cancer Biorepository Network (www.prostatebiorepository.org (accessed on 23 February 2017)). These samples were harvested after patient death, during rapid autopsy allowing to obtain a large number of metastatic samples from the same patient. The second TMA was composed of samples from PCa patients treated at Tours University Hospital (Tours, France), and includes bone metastasis from 13 patients and lymph node metastases from 11 patients. Written informed consents were obtained for all patients in accordance of the requirements of the medical ethic committee. Not all cases embedded in TMAs could be analyzed for each antibody, due to loss of tissue core, core folding, or non-interpretable staining. Immunohistochemistry was performed as previously described [4] using the primary anti-CCR3 antibody (clone Y31, reference ab32512, obtained from Abcam, Cambridge, MA, USA) diluted to 1/100 and incubated overnight at 4 °C. Two pathologists (pathology unit of Tours University Hospital) who were blind to clinical data, independently scored CCR3 expression in *human* tumors as negative or positive staining.

### 4.5. In Silico Meta-Analysis

The Oncomine database (Oncomine TM v. 4.5, www.oncomine.org (accessed on 23 May 2017)) was used for datamining and in silico meta-analysis [49]. We used six published datasets Oncomine (Table 1), including 249 primary sites and 28 bone metastases of interest for RNA analyses. We employed filters for selection of conditions and genes of interest (PCa; bone metastasis; primary site; genes). Data were ordered by ‘overexpression’ and the threshold was adjusted to *p*-value < 0.0001; fold change, 2 and gene rank, top 10%. For each database, only genes that met the criteria for significance were reported.

### 4.6. Statistical Analysis

Statistical analysis was performed using STATA 12 software (StataCorp LLC, College Station, TX, USA) and Prism v. 7 (GraphPad Software, San Diego, CA, USA). Two group comparisons were performed using Mann-Whitney’s test or Student’s t test and multiple comparisons were performed by One-way or two-way ANOVA with the indicated associated post hoc tests. To determine the appropriate post hoc test to apply, normality of samples was determined using Shapiro-Wilk test. For the TMA analysis, both TMA were associated and distributions of CCR3 expression in the three sites (lymph node (LN), visceral (V) and bone metastasis (BM)) were compared using Chi2 distribution comparison. All statistical tests were two-sided. *p* values < 0.05 (*), < 0.01 (**), < 0.001 (***) and < 0.0001 (****). “ns” stand for “not significant”.

## Figures and Tables

**Table 1:** Main characteristics of studies included in meta-analysis.

Author	Year	Microarray	Primary Site (*n*)	Bone (*n*)
Grasso [30]	2012	Agilent Human Genome 44K	59	0
LaTulippe [31]	2002	Human Genome U95A-Av2 Array	23	2
Liu [32]	2015	Human Genome U133A Array	3	13
Ramaswamy [33]	2001	HumanGeneFL Array + Hu35KsubA Array	10	3
Tamura [34]	2007	Platform not pre-defined in Oncomine	23	8
Taylor [35]	2010	Platform not pre-defined in Oncomine	131	2

## Data Availability

The data presented in this study are available on request from the corresponding author.

## Supplementary Materials

The following are available online at https://www.mdpi.com/1422-0067/22/4/1994/s1.
