# Supplementary material for: The Chemokine Receptor CCR3 Is Potentially Involved in the Homing of Prostate Cancer Cells to Bone: Implication of Bone-Marrow Adipocytes
Source: Int J Mol Sci. 2021 Feb 17;22(4):1994. doi: 10.3390/ijms22041994 (PMC7922974; doi:10.3390/ijms22041994)
Supplement: Supplementary file 1 [file ijms-22-01994-s001.pdf]

Figure S1

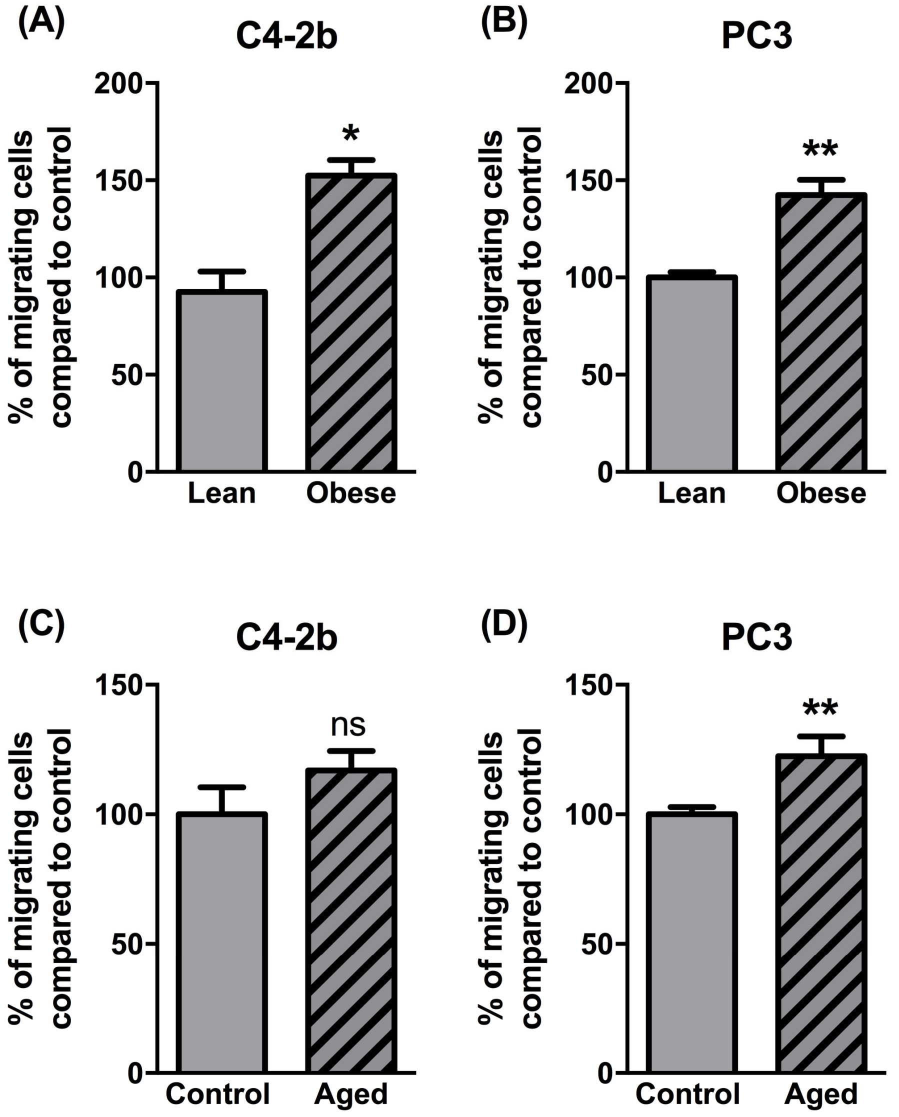

Figure S1. **Supporting Information:**

Figure S1. Regulation of the chemotaxis of PCa cells towards SAT-CM by obesity and ageing.

In vitro migration of C4-2B **(A)** or PC3 **(B)** towards conditioned medium SAT (SAT-CM) from lean (age: 62,8 ± 6,9 years, BMI: 21,4 ± 3,3 kg/m^2) or obese (age: 63,3 ± 10,1 years, BMI: 33,3 ± 2,3 kg/m^2) subjects (n=5/group). *In vitro* migration of C4-2B **(C)** or PC3 **(D)** towards conditioned medium SAT (SAT-CM) of aged (age: 75,7 ± 5,7 years, BMI: 23,3 ± 4,3kg/m^2) or younger control (age: 60,2 ± 5,2 years, BMI; 22,1 ± 3,5kg/m^2) subjects (n=5/group). Data are shown as mean ± sem. Statistical analysis by Student t test was performed. * $p<0.05$, **$p<0.01$, ns: not significant.
